# Breaking the premetastatic niche barrier: the role of endothelial cells and therapeutic strategies

**DOI:** 10.7150/thno.113665

**Published:** 2025-05-25

**Authors:** Yingshuai Fang, Wenming Cui, Yabing Yang, Xinhao Zhang, Mengyao Tian, Zhiyuan Xie, Ying Guo, Weitang Yuan, Zhen Li, Shuaixi Yang

**Affiliations:** 1 The First Clinical School of Medicine, Zhengzhou University, Zhengzhou 450001, China.; 2 Department of Colorectal Surgery, the First Affiliated Hospital of Zhengzhou University, Zhengzhou 450000, China.

**Keywords:** Endothelial cells, Immunosuppression, Premetastatic niche, Targeted therapy, Angiogenesis

## Abstract

The premetastatic niche (PMN) represents a metastasis-facilitative microenvironment established prior to tumor dissemination, initiated by vascular leakage and endothelial cell (EC) functional remodeling. ECs play pivotal roles as bridges in different stages of the metastatic cascade. As critical stromal components within the PMN, ECs not only drive angiogenesis but also actively orchestrate immune suppression, extracellular matrix (ECM) remodeling, and the inflammatory signaling characteristic of PMN formation, with multiple specific signaling pathways such as VEGF/Notch playing a crucial role. With the evolving understanding of the role of ECs in controlling tumor metastasis, therapeutic strategies targeting ECs within the PMN, such as antiangiogenic therapy (AAT), targeting of endothelial glycocalyx (GCX), inhibition of tumor-derived exosome (TDE) and angiocrine signaling, are becoming research hotspots. This review systematically delineates the cellular and molecular composition of PMNs, dynamically dissects their spatiotemporal evolution, and highlights organ-specific mechanisms of EC-driven PMN establishment. Furthermore, we summarize emerging EC-targeted therapeutic strategies, providing innovative insights for inhibiting tumor metastasis.

## Introduction

Tumor metastasis remains the leading cause of cancer-related mortality. Despite recent advancements in cancer therapeutics, metastatic cancers continue to pose significant clinical challenges 1. A critical step in metastasis involves the shedding of circulating tumor cells (CTCs) from primary tumors, which disseminate through hematogenous or lymphatic systems to colonize distant organs 2. The organotropism of metastasis can be attributed to specialized microenvironments that actively recruit tumor cells (TCs), as conceptualized by Paget's 1889 “seed and soil” hypothesis, which emphasizes bidirectional interactions between TCs (seeds) and host organs (soil) 3. This metastasis-permissive microenvironment was later termed the PMN by Lyden et al. 4. Emerging evidence reveals that tumors precondition distant organs by establishing PMNs prior to metastatic colonization 5. Characterized by immune suppression, angiogenesis, vascular hyperpermeability, and organotropism, PMNs provide a hospitable ecosystem for TCs 5. Consequently, PMN biology has garnered increasing recognition as a pivotal determinant of metastatic efficiency.

ECs, as active participants in the tumor microenvironment (TME), play a key role in angiogenesis and cancer progression. In addition to their canonical angiogenic function in supplying nutrients, ECs facilitate TC extravasation through vascular permeability modulation and immune evasion 6. Moreover, TCs play a decisive role in promoting metastasis by promoting angiogenesis at distant sites 7. ECs are uniquely positioned to orchestrate interactions among stromal, immune, and molecular components, distinguishing them from other cell types. Unlike other stromal cells, ECs directly shape the immune landscape through cytokine/chemokine secretion (e.g., IL-6, CCL2), which regulates immune cell trafficking and polarization. The unique ability of ECs to interact with immune and stromal components is further emphasized in ECM remodeling: post-translational modifications of ECM components dynamically regulate EC adhesion and paracrine signaling, thereby influencing cellular spatial organization within the TME 8. Additionally, the cellular composition of the TME varies greatly among different tumor types, with ECs playing a central role. ECs induce the expression of tight junction proteins by expressing specific transcription factors (e.g., ETS1 and SOX7), enhancing the integrity of intercellular barriers, and thereby affecting the TME 9. This review systematically examines the cellular and molecular composition of PMNs, with a particular emphasis on the multifaceted roles of ECs in PMN establishment. We further highlight the differential effects of ECs across distinct metastatic organs. Finally, we synthesized emerging therapeutic strategies targeting PMN-associated ECs, aiming to provide novel insights for preventing metastatic progression.

## PMN: The Soil for Tumor Metastasis

Over the past decade, our understanding of the PMN has deepened significantly, particularly with respect to the multifaceted roles of the immune system, stromal cells, tumor-derived secretory factor (TDSFs), and miRNA-enriched extracellular vesicles (EVs) in PMN dynamics. This progress has propelled PMN research from fundamental exploration toward clinical translation for preventing and treating metastatic progression. Here, we delineate the developmental stages and formation mechanisms underlying PMN establishment.

### Cellular Constituents: Stromal-Immune Synergy

#### Stromal cells

The formation of the PMN involves intricate interactions among stromal cells, the vasculature, and the ECM, which are dynamically remodeled during crosstalk with primary tumors 10. Stromal cells facilitate metastatic progression by secreting chemokines (e.g., CCL2) to recruit myeloid-derived suppressor cells (MDSCs) and tumor-associated macrophages (TAMs) 11. Fibroblasts play pivotal roles in PMN establishment and therapeutic resistance 12. For example, ovarian cancer cells extensively secrete exosomes enriched with miR-141, which activate the YAP1/GROα/CXCR signaling cascade to mediate tumor-fibroblast interactions, thereby fostering PMN development 13. Moreover, pericytes within PMNs also exhibit protumorigenic properties. Murgai et al. demonstrated that tumor-derived factors induce KLF4 expression in pericytes, which results in the formation of fibronectin-rich PMNs, whereas conditional KLF4 knockout in pericytes suppresses their expansion and impedes lung metastasis 14. Notably, organ-specific stromal responses occur—pulmonary ECs downregulate tumor necrosis factor-related apoptosis-inducing ligand (TRAIL) under VEGF stimulation to promote PMN formation, whereas hepatic sinusoidal ECs increase fibronectin expression via TGF-β1 to drive tumor progression 15, 16. Therefore, an in-depth exploration of stromal cell functions within PMNs has significant implications for the development of effective strategies to improve cancer therapeutics.

#### MDSCs

MDSCs, a heterogeneous population of immature myeloid cells with immunosuppressive functions, directly drive tumor metastasis by participating in PMN formation, promoting angiogenesis, and enhancing tumor invasion 17, 18. Phenotypically, MDSCs are categorized into polymorphonuclear (PMN-MDSC) and monocytic (M-MDSC) subsets, which morphologically resemble neutrophils and monocytes 19. Studies have demonstrated that the NLRP3-HSP70 axis in melanoma induces PMN-MDSC accumulation in lung tissues via TLR4 signaling-dependent mechanisms in pulmonary epithelial cells, fostering PMN development and conferring immunotherapy resistance 20. Before TCs arrive, lung-infiltrated M-MDSCs enhance TC adhesion to ECs in the PMN by secreting IL-1β to upregulate ECs E-selectin expression 21. Notably, specific TDSFs (e.g., TIMP-1 and macrophage migration inhibitory factor [MIF]) facilitate hepatic PMN formation through MDSC recruitment 22. Chronic psychological stress activates the glucocorticoid-TAM-CXCL1 axis, driving splenic MDSC mobilization to construct breast cancer PMNs via CXCR2 signaling 23. Mechanistically, galectin-1 recruits PMN-MDSCs through STING activation to mediate ECM remodeling, whereas PMN-MDSCs increase CTC metastatic potential via ROS/Notch/Nodal signaling crosstalk 24. Thus, MDSC accumulation orchestrates PMN maturation and pulmonary metastasis progression.

#### Neutrophils

Neutrophils, the most abundant immune cells in peripheral blood, have garnered significant attention for their dual regulatory roles in tumor progression 25. Lin28B, nicotine, and TLR3 drive the recruitment of N2-polarized neutrophils to establish an immunosuppressive PMN during breast cancer lung metastasis 26-28. Neutrophil phenotypic polarization is dynamically regulated by microenvironmental cues: IFN-1 induces antitumor N1 polarization, whereas TGF-β suppresses N1 differentiation and promotes protumor N2 polarization, with the latter secreting arginase 1 to deplete arginine and impair T-cell cytotoxicity 29, 30. The recruitment mechanism of neutrophils involves various signal molecules such as chemokines (e.g., CXCL1/8), exosomes, and bioactive factors. Notably, TANs dynamically interact with CTCs through adhesion molecule engagement, cytokine secretion, and neutrophil extracellular trap (NET) formation, thereby modulating the PMN and TME remodel. Experimental evidence confirms that SPP1-induced NETs facilitate hepatocellular carcinoma lung colonization by trapping CTCs 31, whereas strategies targeting CTC‒neutrophil interactions (e.g., biomimetic nanoparticle blockade) exhibit antimetastatic potential 32, 33. Mechanistically, NETs degrade the ECM via protease release (e.g., MMP9) and reactivate dormant TCs, underscoring their critical role in metastatic cascades 34. In summary, the interaction between neutrophils and ECs is critical for the migration of TCs and the formation of immunosuppressive PMN, as we will describe in further detail in the following sections 35-39.

#### Macrophages

TAMs dynamically shift their transcriptional programs along a continuous spectrum influenced by TME-derived stimuli, with M1 (antitumor) and M2 (protumor) phenotypes representing polar extremes 40. The M2 subset dominates PMN establishment by orchestrating immune suppression and angiogenesis 41. M1 TAMs are typically activated by LPS and IFN-γ, while M2 TAMs are activated by cytokines such as IL-4 and IL-13 42. Intermediate macrophage phenotypes (M2a, M2b, M2c, M2d) have been identified and described, and they significantly impact PMN formation and EC behavior 43. For instance, M2a macrophages promote angiogenesis, whereas M2b macrophages are involved in immune cell recruitment and inflammatory responses 44. TDEs deliver specific molecules (e.g., miR-934 and miR-4488) or regulate critical pathways (CAP2-mediated TGF-β1 secretion; Caveolin-1/PTEN-CCL2/VEGF-A axis) to drive M2 polarization, thereby enhancing hepatic/pulmonary metastasis and vascular remodeling 45-48. Notably, distinct macrophage subsets predict divergent clinical outcomes, highlighting the importance of resolving TAM heterogeneity. Recent single-cell studies classify TAMs into resident tissue macrophages (RTMs) and monocyte-derived TAMs 42, 49: RTMs activate fibroblasts and induce immunosuppression via phagocytosis of TDEs, whereas monocyte-derived TAMs remodel the PMN through proinflammatory cytokine secretion. This spatial-temporal regulatory mechanism underscores the therapeutic potential of targeting TAM subset differentiation.

#### T and B lymphocytes

As core components of adaptive immunity, T/B lymphocytes play complex regulatory roles within the tumor immune microenvironment. PMN establishment is closely associated with regulatory T-cell (Treg)-mediated immunosuppression 50. Tregs shape CTC-disseminating microenvironments via the secretion of cytokines (e.g., TGF-β and IL-10), with hepatic infiltration of TNFR2+ Treg subsets correlating with poor prognosis in lung/colorectal cancer liver metastasis 51, 52. Pharmacological inhibition of Tregs through NEDD8 pathway targeting effectively reduces postoperative pulmonary metastasis in patients with colorectal cancer 53. Th2 polarization accelerates metastatic progression via STAT6-dependent complement C3 upregulation (driving neutrophil recruitment and NET formation) and macrophage phenotypic reprogramming 54, 55. Notably, exosomal miR-135a-5p directly suppresses CD4+ T-cell activation, fostering liver metastasis immune tolerance 56.

B lymphocytes regulate the PMN through antibody-independent mechanisms, exhibiting unique roles in lymph node-breast cancer immune crosstalk 57. In breast cancer, B cells drive lymph node metastasis via the HSPA4 glycosylation-IgG-CXCR4/SDF1α axis, while single-cell sequencing revealed that transcriptional reprogramming of marginal zone B cells in tumor-draining lymph nodes (angiogenic pathway activation) is negatively associated with prognosis 2, 58. Intriguingly, T/B-cell synergism promotes breast cancer bone metastasis, although their spatiotemporal regulatory dynamics in the PMN remain incompletely characterized 59. The current understanding of T/B lymphocyte interactions within the PMN remains limited; thus, conducting more comprehensive studies on these two types of cells is necessary.

### Molecular Drivers: Dual Regulation by TDSFs and EVs

The formation of PMNs involves intricate interactions between diverse cellular and molecular components, which collectively orchestrate PMN development. These molecules originate not only from bone marrow-derived and stromal cells but also from TCs. While the previous sections focused on the roles of bone marrow-derived immune cells and stromal cells in PMN establishment, tumor-derived molecular components are pivotal in driving organ-specific PMN formation. Therefore, we focused on TDSFs and extracellular vesicles.

#### TDSFs

TDSFs include proteins, enzymes, cytokines, and other bioactive molecules secreted by TCs under hypoxic and inflammatory conditions, exerting critical regulatory effects on tumor progression and PMN establishment. The functional roles of TDSFs vary across tumor types, with accumulating evidence demonstrating that TDSFs promote PMN formation by mobilizing and recruiting bone marrow-derived cells. For example, in colorectal cancer, TDSFs recruit MDSCs via the S1PR1-STAT3 signaling axis to facilitate hepatic PMN formation 60^.^ Additionally, CCL2-mediated recruitment of Tregs and TAMs stimulates angiogenesis and immunosuppression, fostering pulmonary PMN development 61, 62. Recent studies have revealed that gastric cancer-derived lipopolysaccharide-binding protein activates the TLR4/NF-κB pathway in hepatic macrophages and hepatic stellate cells, driving fibrotic PMN formation in the liver 63. In summary, TDSFs interact with bone marrow-derived immune or host stromal cells through distinct signaling pathways, thereby inducing these cells to secrete specific molecular components to support PMN formation.

#### EVs

Tumor-derived EVs, pivotal mediators of TME crosstalk, exert pleiotropic regulatory effects on tumor growth and metastatic cascades by delivering bioactive cargoes, including nucleic acids, proteins, and metabolites 64, 65. Classified by biogenesis pathways, EVs include exosomes, microvesicles, apoptotic bodies, oncosomes, and megasomes, with exosomes and microvesicles being extensively studied for their roles in PMN formation 66. In recent years, mechanistic insights into EV-mediated metastasis and PMN establishment have expanded, notably identifying tumor exosomal integrins as predictive biomarkers for organotropic metastasis 67-70. miRNAs are identified as early drivers of PMN driven by EVs, and are involved in regulating nearly all cancer-associated processes, including ECM remodeling, angiogenesis, and immune cell recruitment 71, 72. For instance, miR-21 targets PTEN and the Akt signaling pathway, reducing the proliferation of Tregs and thereby modulating the function of immune cells 73. miR-29a/c targets VEGF to inhibit angiogenesis in the gastric cancer microenvironment 74. Additionally, in colorectal liver metastasis, circ-0034880-enriched EVs increase the activation of SPP1 high CD206^+^ protumorigenic macrophages, remodeling the host stromal microenvironment to foster overt metastasis 75. Recent studies have focused on understanding the effects of specific factors on EV properties and function. For example, Snail overexpression in murine colon adenocarcinoma increases Glypican-1 levels in EVs, potentially augmenting PMN development and metastatic potential 76. Notably, studies in xenograft models revealed that tumor EVs prime inflammatory responses in distant organs, accelerating PMN maturation 77.

### Dynamic evolution of the PMN: Spatiotemporal orchestration by TCs

TCs establish permissive microenvironments in distant organs through spatiotemporally coordinated mechanisms to enable metastasis. As a critical metastatic checkpoint, PMN formation evolves through three dynamically interconnected phases.

**Phase I: Molecular preprogramming.** Primary tumors remotely precondition potential metastatic organs via the systemic secretion of exosomes, cytokines, and other signaling molecules. These tumor-derived components (e.g., miRNAs and integrins) establish organotropic molecular imprints through hematogenous/lymphatic circulation, systematically preconfiguring the PMN landscape. Recently, Wang et al. systematically organized the roles and mechanisms of known tumor-derived molecular components in PMN formation, which provided a novel understanding of PMN formation 78.

**Phase II: Microenvironmental remodeling.** Resident organ cells respond to tumor-derived signals by initiating angiogenesis, immune evasion, and ECM remodeling to prime metastatic colonization. For example, KLF4-dependent perivascular cells mediate angiogenesis and ECM remodeling to promote PMN maturation 14. Concurrently, cellular and molecular components within PMNs enhance TC invasiveness: tumor-associated neutrophils (TANs) facilitate CTC-endothelial adhesion during intravasation, whereas TANs directly interact with CTCs to increase their survival 79-82. Hippo pathway inactivation further amplifies CTC aggressiveness via Wnt/β-catenin-mediated epithelial-mesenchymal transition (EMT), as evidenced by HCC EVs delivering miR-665/miR-1273f to suppress Hippo signaling and increase CTC stemness 83-85. Through these spatiotemporal synergies, tumors engineer premetastatic soil long before physical CTC arrival 86.

**Phase III: CTC Homing and Expansion.** Mature PMNs guide CTC homing through chemotactic gradients. Bidirectional CTC‒PMN crosstalk critically regulates colonization: nicotine-induced N2 TANs activate CTC EMT via adiponectin 2, whereas CTC-derived IL-6 drives neutrophil N2 polarization through STAT3 signaling, forming a prometastatic feedforward loop 28, 87-89. PMN-MDSCs further enhance CTC metastatic fitness through ROS/Notch crosstalk 24. Postcolonization, CTCs either enter dormancy or exploit remodeled niches for proliferation, ultimately developing macroscopic metastases.

**Figure 1:**
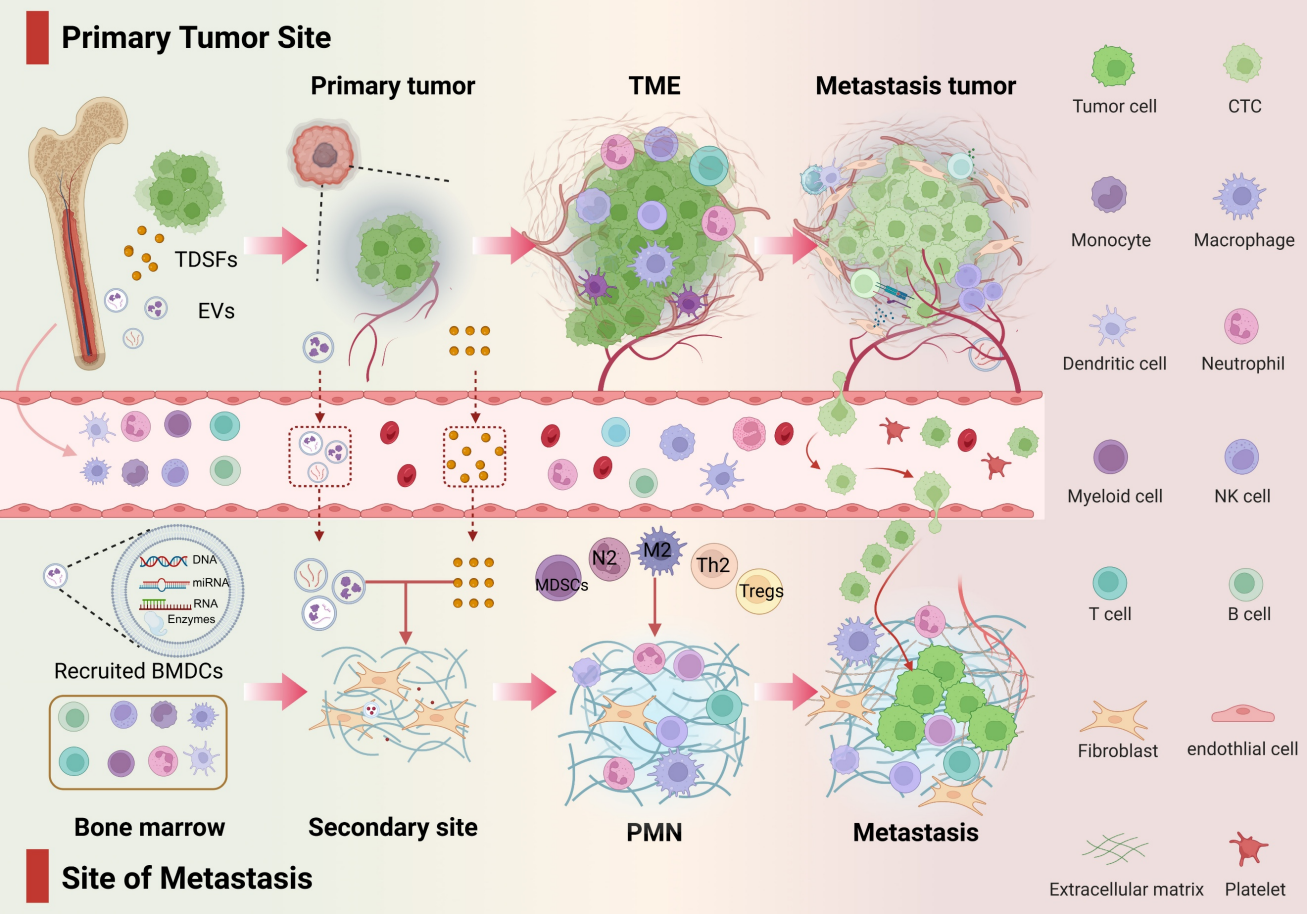
** Developmental stages of the PMN.** From the primary tumor to PMN, TCs promote metastasis in a spatiotemporal manner. This figure outlines the three-stage dynamic evolution of PMN from initiation to maturation. The entire process reflects the precise orchestration by TCs of the preparation of the “soil” in distant organs and their own colonization. **Phase I:** Molecular preprogramming—Primary tumors release exosomes, cytokines, and other factors to remotely regulate target organs, establishing chemotactic gradients and vascular leakage signatures. **Phase II:** Microenvironment remodeling—Resident cells in the target organ respond to tumor signals, constructing the PMN through angiogenesis, immune suppression, and ECM remodeling. **Phase III:** CTC Homing and Expansion—CTCs interact with the modified microenvironment, achieving metastatic focus formation through mechanisms such as immune evasion and stemness activation.

Progressive CTC colonization of mature PMNs drives pathological progression from micrometastases to macrometastases, with CTC-laden PMNs potentially promoting further dissemination. Collectively, PMN evolution reflects not stochastic events but a precision-engineered spatiotemporal program wherein tumors precondition distant microenvironments to license metastatic outgrowth (Figure 1).

## EC contributions to PMN formation

**Figure 2:**
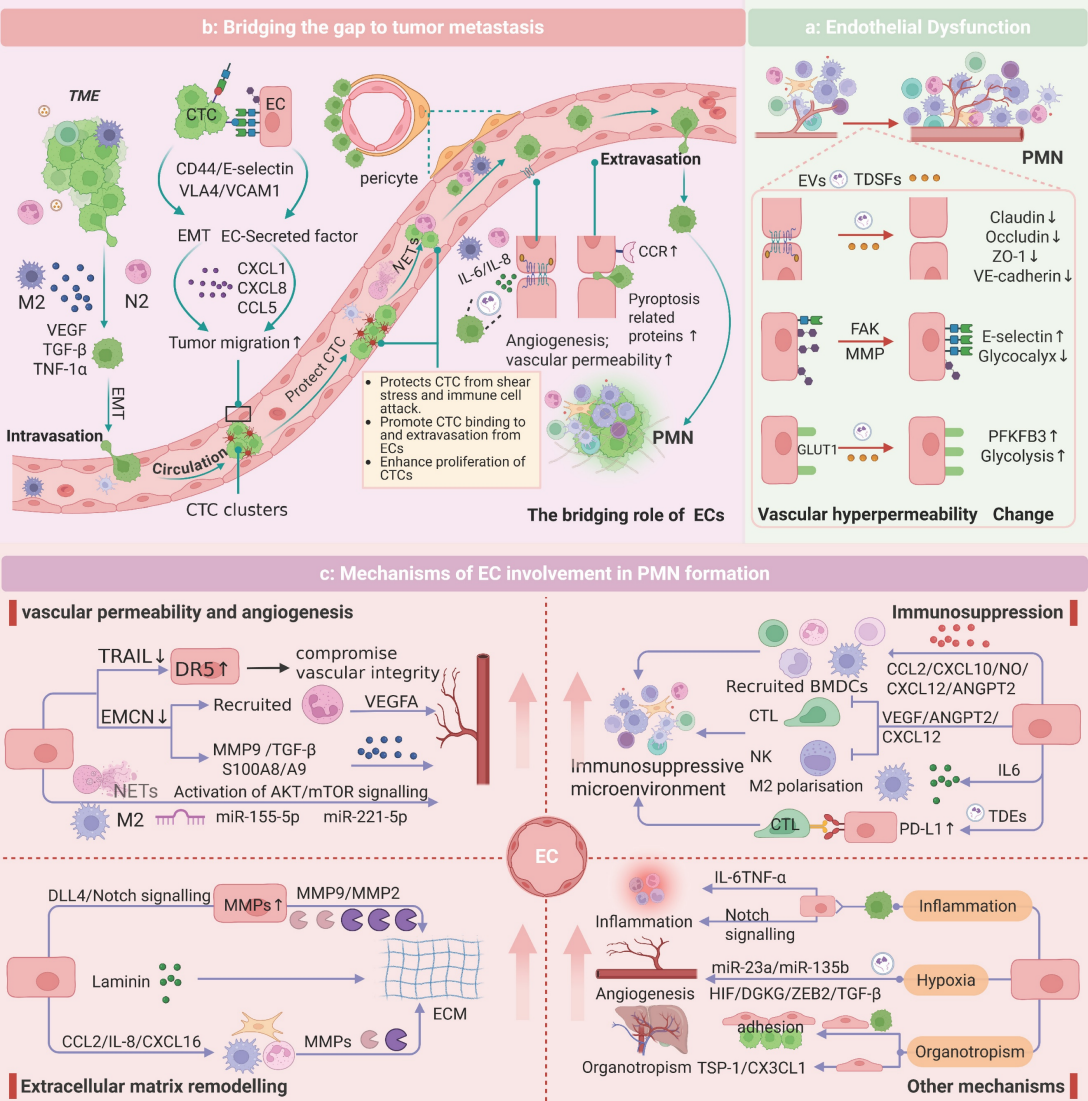
** Endothelial Mechanisms in PMN Evolution. a:** In the formation of the PMN, TDSFs and EVs induce endothelial dysfunction, compromising vascular barrier integrity. This manifests as increased vascular permeability and leakage. The underlying mechanisms involve multiple factors, including disruption of adherens junctions, activation of MMPs, degradation of the GCX, and metabolic reprogramming. **b:** ECs play a pivotal role in every stage of tumor metastasis. By modulating adhesion molecules, secreting chemokines, and maintaining the dormancy of CTCs, ECs facilitate the intravasation, survival in circulation, extravasation, and colonization of CTCs. ECs also provide a “bridge” for TCs and immune cells, creating a microenvironment conducive to metastasis. **c:** ECs contribute to the development of PMN characteristics, with recruited immune cells and tumor-derived molecular components interacting with ECs via the vasculature to prepare a mature environment for tumor metastasis within the PMN.

The PMN represents an aberrant, tumor-permissive microenvironment devoid of cancer cells. Previous studies have indicated that PMN formation initiates with localized alterations (e.g., vascular leakage and stromal/ECM remodeling) before exerting systemic immunosuppressive effects. ECs, as pivotal PMN components, actively participate in niche establishment through immune suppression, inflammation, angiogenesis, and ECM remodeling. Here, we aimed to reveal the specific role of ECs in PMN formation by performing PMN studies related to ECs (Figure 2).

### Stage-Specific Functions of ECs in the PMN

#### Endothelial Dysfunction: Vascular Barrier Disruption

Under physiological conditions, ECs maintain vascular integrity via VE-cadherin-mediated adherens junctions and occludin/claudin/ZO-1 tight junction complexes 90. During PMN formation, TDSFs and EVs destabilize endothelial barriers, inducing pathological hyperpermeability 91. For example, colorectal cancer-derived EVs (e.g., ADAM17 and miR-27b-3p) synergistically promote vascular leakage by interfering with the membrane localization of VE-cadherin and regulating the expression of tight junction proteins (ZO-1/occludin/Claudin5) via the KLF2/KLF4-VEGFR2 axis 92-94. Breast cancer-derived angiopoietin 2 exacerbates junctional instability by activating MMPs 95. Concurrently, GCX cleavage enzymes (e.g., heparanase, MMPs) in the PMN increase permeability via syndecan-3/4 ectodomain cleavage in a Rho-kinase-dependent manner 96-98. Metabolic reprogramming also contributes: VEGF-enriched EVs drive endothelial hyperglycolysis via PFKFB3/GLUT1 upregulation, increasing leakage 99. Increased vascular permeability facilitates bidirectional cellular trafficking and microenvironmental crosstalk, thereby accelerating PMN formation (Figure 2a).

#### Microenvironment remodeling: Endothelial-stromal-immune cross talk

ECs are driven by tumor-derived molecules and work together with stromal and immune cells to shape PMNs. On the one hand, stromal and immune cells within the PMN regulate EC functions via paracrine signaling. Fibroblasts activate the P38 MAPK pathway in ECs via the lncRNA SNHG5 to promote angiogenesis 100. M2-polarized TAMs secrete exosomes enriched with miR-23a/155/221 to induce vascular leakage 101, 102, whereas interstitial macrophages increase vascular permeability through the IL-6/VEGF axis 103. On the other hand, ECs autonomously contribute to the construction of an immunosuppressive microenvironment and ECM remodeling. For instance, tumor-derived autophagosomes upregulate PD-L1 expression on ECs via the TLR4-MyD88-p38/STAT3 cascade, suppressing T-cell activity and polarizing macrophages toward the M2 phenotype 104, 105. Activated Notch1 receptors (N1ICDs) in ECs drive neutrophil infiltration and metastasis 39. Additionally, CD36 upregulation in ECs exacerbates immunosuppression 106. This reciprocal crosstalk between ECs and neighboring cells is highly important for the establishment of a mature PMN, which primes the microenvironment for subsequent TC dissemination and colonization.

#### Metastatic cascade regulation: endothelial-mediated CTC homing and extravasation

ECs serve as “bridges” for TCs and immune cells during the metastatic process. In the initial stage of metastasis, TAMs release EGF/TNF-α to promote the intravasation of TCs, and TANs guide TCs to the vascular endothelial interface through chemotaxis 80, 107. CTCs in circulation face shear stress and immune clearance pressures, and their survival and metastatic efficiency are precisely regulated by adhesion molecules on the endothelial surface 82, 108. E-selectin mediates transendothelial migration by binding to the CD44 receptor on the surface of CTCs, and its expression is positively regulated by IL-1β secreted by M-MDSCs 21, 109. ECs in the leaky region form microenvironmental “hotspots” for metastatic cancer cell-specific homing through focal adhesion kinase (FAK)-dependent upregulation of E-selectin 110, 111. For example, E-selectin is an important homing receptor for hematogenous dissemination in lung cancer, prostate cancer, and breast cancer 112-114. In bone, E-selectin can also promote the EMT of CTCs, increasing bone metastasis. VCAM1 and ICAM1 are also key adhesion molecules that drive lung/breast-specific colonization of CTCs through interactions with VLA4 and integrins 115, 116. Additionally, ECs reduce immune cell adhesion by suppressing VCAM-1/ICAM expression and form a physical barrier to protect CTCs from immune surveillance through platelet aggregation 117, 118. Chemokines secreted by ECs (such as CXCL1/CXCL8/CCL5) guide the directional migration of CTCs through gradients, with CCL5 activating the androgen receptor to increase the invasiveness of prostate cancer 119, 120. Molecules such as Biglycan and EphrinA1 also play roles in promoting TC migration 121. Studies on pericytes have shown that CTCs replace pericytes by competing for L1CAM on ECs, thereby achieving vascular basement membrane infiltration 122. During extravasation, the expression of CCR2 on ECs leads to endothelial retraction and TC extravasation 123. Recently, high expression of pyroptosis-related proteins in ECs was shown to further accelerate this process 124. Notably, ECs maintain the dormancy of CTCs through factors such as thrombospondin-1, providing a potential niche for metastatic relapse 91, 114 (Figure 2b).

In summary, ECs play a unique role in the formation of the PMN by altering their function, remodeling the microenvironment, and providing a “bridge,” thereby laying the foundation for CTC colonization. We also summarize the relevant molecular mechanisms by which TDEs target ECs to promote the formation of the PMN (Table 1).

### Core mechanisms of EC involvement in PMN formation

#### Angiogenesis

As discussed, ECs critically regulate vascular permeability and TC migration. Angiogenesis, as one of the core mechanisms of PMN formation, is orchestrated by ECs through multiple pathways. For instance, EMCN-deficient ECs recruit Ly6G+ neutrophils and upregulate MMP9, S100A8/A9, and TGF-β to induce proangiogenic phenotypes and pulmonary PMN formation 35. In breast cancer models, loss of TRAIL expression activates the death receptor DR5, triggering NF-κB/p38-dependent adhesion phenotype switching in ECs to promote myeloid cell infiltration and tumor colonization 15.

On the other hand, M2 macrophage-derived exosomal miRNAs (e.g., miR-155-5p and miR-221-5p) regulate endothelial migration and angiogenesis by targeting molecules such as GJA1, whereas miR-30a-5p reprograms EC function via PDCD10-dependent mechanisms 152-154. Neutrophils amplify angiogenesis via JAK/STAT3-mediated VEGFA activation in combination with G-CSF signaling, and NET-DNA enhances this effect by binding the ccdc25 receptor on HUVECs to activate the AKT/mTOR pathway 155, 156. Notably, exosomal ANGPTL1 imposes vascular hyperpermeability and delays PMN maturation by reprogramming Kupffer cells and suppressing MMP9 expression 157. In addition to TANs, the angiogenic mechanisms in PMNs involving diverse cellular and molecular components have been systematically elucidated.

#### Immunosuppression

Immune suppression is a well-recognized facilitator of PMN formation, in which ECs drive the immunosuppressive characteristics of the PMN through multiple mechanisms. EVs reprogram ECs to facilitate immunosuppressive cell infiltration and functional polarization 146. Activated ECs mediate immune cell transendothelial migration by secreting chemokines (e.g., CCL2 and CXCL10), while IL-6 secretion drives macrophage polarization toward protumor phenotypes 158, 159. Recently, the CXCL12+ EC subpopulation was shown to establish an HCC-specific immune escape microenvironment by inhibiting cytotoxic T lymphocyte activity and recruiting MDSCs 160.

Moreover, proangiogenic molecules such as VEGF induce immune exhaustion by increasing PD-1/CTLA-4 expression on Tregs and CD8+ T cells 161. VEGF also suppresses dendritic cell activation, thereby impairing T-cell priming 162. Under inflammatory stimuli, angiopoietin-2 (ANGPT2) synergizes with TNF-α to recruit Tregs/MDSCs via adhesion molecule modulation, amplifying immunosuppression 163, 164. In breast cancer, tumor-derived autophagosomes activate the TLR4‒MyD88 signaling pathway in ECs to upregulate PD-L1 expression, directly inhibiting T-cell function 105. These findings collectively highlight ECs as pivotal regulators of immune dynamics, coordinating spatiotemporally resolved molecular networks to establish immunosuppressive niches within PMNs.

#### ECM Remodeling

The ECM, a complex network of proteins and glycosaminoglycans, plays a pivotal role in TC motility and invasion. TDSFs remodel PMN matrix stiffness and topology by regulating the expression of ECM structural proteins (e.g., laminin), degradative enzymes (MMP family), and processing proteins 165. Mechanistically, ECs directly cleave ECM components via MMP-2/MMP-9 and activate stromal cell MMP secretion through paracrine cytokines such as CCL2/IL-8 166. Additionally, the activated DLL4/Notch signaling axis upregulates endothelial MMP9 expression, whereas neutrophil-derived MMPs disrupt vascular integrity by degrading VE-cadherin, synergistically facilitating CTC extravasation 167, 168.

Notably, tumor-specific ECM remodeling features significantly impact clinical outcomes. Melanoma ECs secrete laminin to drive invasive phenotypes, while elevated laminin expression in renal cell carcinoma is correlated with poor prognosis 169. Prior studies using glioblastoma 3D bioprinted cultures containing TCs, ECs, and hyaluronic acid derivatives demonstrated how ECM stiffness modulates transcriptional programs and tumor‒endothelial crosstalk 170. In summary, endothelium-mediated ECM remodeling provides both physical scaffolding and chemotactic gradients to support tumor metastasis.

#### Other mechanisms: inflammation, hypoxia, and organotropism

**Inflammation:** Chronic inflammation drives PMN formation via endothelial dysfunction 171. ECs mediate tumor-endothelial interactions through ICAM-1, triggering IL-6/TNF-α inflammatory cascades to establish liver-metastatic microenvironments 172. Notch signaling activation induces endothelial senescence, amplifying neutrophil infiltration and proinflammatory cytokine secretion to accelerate tumor adhesion and metastasis 38. EC-derived CCL5 recruits monocytes to promote breast cancer dissemination 173, whereas macrophage‒endothelial crosstalk exacerbates inflammatory responses through hypoxia-dependent mechanisms 174. Additionally, IGFBP7hi endothelial subpopulations disrupt GCX integrity, exposing adhesion molecules to facilitate T-cell extravasation and amplify inflammatory microenvironments 175. These findings highlight ECs as pivotal orchestrators of inflammatory niche establishment.

**Hypoxia:** Hypoxia, a central driver of angiogenesis, coordinates PMN formation through differential endothelial regulation of hypoxia inducible factors (HIFs) 176. Dynamic HIF-1α/HIF-2α expression under acute vs. chronic hypoxia reshapes lung metastatic niches, enhancing TC dissemination 176, 177. HIF signaling induces endothelial-specific DGKG expression, activating the ZEB2/TGF-β1 axis to promote proangiogenic phenotypes and Treg differentiation 178. Hypoxia-derived exosomal miR-23a/135b regulates PMN maturation by targeting vascular permeability and angiogenesis pathways 138, 140. Notably, sarcoma-derived hypoxia-modified collagen VI disrupts pulmonary endothelial barrier integrity, providing structural support for metastasis 179.

**Organotropism:** ECs critically regulate organ-specific metastasis. Transfer of tumor-derived microRNAs to ECs modulates their migratory properties and organ selectivity 180. The causative role of blood flow is a key factor in the direct regulation of organ-specificity by the vasculature. For example, the liver's unique blood supply pattern can cause primary tumors to metastasize to the liver through the portal vein. Hemodynamics and fluid flow patterns not only facilitate the transport of CTCs but also influence their ability to colonize distant sites, as evidenced by the patterns observed in liver and lung interactions 181. Moreover, the regulation of organotropism is multifaceted, involving various biological factors, including the intermittent nature of blood flow in specific vascular structures, such as liver sinusoids 182. This intermittent flow can affect the survival and colonization potential of metastatic cells, as they must adapt to the changing hemodynamic conditions within the circulatory system 183. CTC-EC adhesion determines organotropism, with TSP-1 and CX3CL1 secreted by ECs influencing TC self-renewal and immune cell recruitment, respectively, to shape “congenial soil” for metastasis 182, 184, 185. Interestingly, mitochondrial transfer from ECs to TCs via tunneling nanotubes enhances invasiveness through metabolic reprogramming 186. Understanding these EC-tumor interactions is vital for deciphering organ-specific metastatic mechanisms 187 (Figure 2c).

## Role of ECs in organ-specific PMNs

Clinical evidence highlights that most cancers metastasize to specific organs, a phenomenon termed organotropism. Investigating the shared and divergent mechanisms of PMNs across metastatic organs is critical for deciphering organotropic metastasis and developing targeted therapies. This section discusses EC-driven mechanisms in organ-specific PMN landscapes.

### Lymph node

Lymph nodes (LNs), as critical hubs of the lymphatic system, favor PMN formation by providing tumor-permissive microenvironments that enhance TC immune evasion and invasiveness. LN-associated PMNs are characterized by lymphangiogenesis and high endothelial venule remodeling 188. TDEs promote characteristic changes in the PMN by remodeling lymphatic endothelial function. For example, melanoma-derived NGFR-enriched EVs are internalized by LECs, activating ERK/NF-κB signaling and upregulating ICAM-1 to increase lymphangiogenesis and TC adhesion 189. ITGA6+ EVs deliver circRNA-LIPAR into LECs, triggering E-selectin-mediated lymphatic remodeling 190. Tumor EVs also coordinate immunosuppression during LN remodeling via interactions with LECs 191. LEC-derived CXCL8 recruits TANs to form NETs, whereas CD36-dependent immune checkpoint signaling reinforces immunosuppression 106, 192. POSTN deposition amplifies lymphangiogenesis via VEGF-C upregulation, facilitating tumor colonization 193. Macrophage S1PR1/NLRP3/IL-1β signaling synergizes with the dendritic cell COX-2/EP3/SDF-1 pathway to increase lymphangiogenesis and PMN maturation 194, 195. Collectively, these findings underscore the intricate crosstalk among ECs, LNs, and PMN formation in metastasis.

### Lung

The high vascular perfusion and oxygen-rich microenvironment of the lungs foster a unique PMN. Tumor-derived EVs (e.g., miR-25-3p, ADAM17, and miR-27b-3p) promote lung PMN vascularization by targeting ECs 92-94. Breast cancer-derived autophagosomes activate the TLR4‒MyD88‒p38/STAT3 cascade in ECs to upregulate PD-L1, suppressing T-cell immune function and facilitating pulmonary metastasis 104, 105. Interestingly, chemotherapy-induced ANXA6+ EVs remodel ECs into prometastatic phenotypes via NF-κB activation 196. Zhang et al. demonstrated that EMCN-deficient ECs (genetically engineered in murine models of breast and lung adenocarcinoma) recruit Ly6G+ neutrophils and increase MMP9, S100A8/A9, and TGF-β expression to drive lung PMN formation 35.

Recent insights highlight LAT1 as a regulator of EC proliferation and VEGF-A/mTORC1-driven angiogenesis, while LAT1 inhibitors suppress lung metastasis by inducing vascular normalization 197, 198. Notably, EC‒EC-neutrophil crosstalk enhances neutrophil transendothelial migration through S100A6-mediated tight junction disruption and TRPM2-dependent VE-cadherin phosphorylation 36, 37. In addition, alveolar epithelial cells modulate endothelial barrier integrity via Wnt/β-catenin signaling, whereas M2 macrophages promote vascular leakage through TGF-β1-induced EMT 199-201. In short, the unique microenvironment of the lungs helps to generate PMNs.

### Liver

ECs are pivotal components of the hepatic microenvironment and critically regulate liver homeostasis and disease pathogenesis 202. EV delivery of von Willebrand factor (VWF) enhances angiogenesis in HCC via a VEGF-A/FGF2-FGFR4/ERK1 positive feedback circuit 145. Recent studies revealed that miR-605-3p suppresses vascularization in hepatic PMNs by reducing exosomal NOS3 levels 7. Experimental evidence has demonstrated that exercise training mitigates liver metastasis susceptibility by inhibiting NET formation and modulating EC adhesion molecule expression 203.

Macrophages are key contributors to hepatic PMN establishment. ECs recruit CX3CR1+ macrophages through CX3CL1 secretion, driving MMP9 upregulation and liver metastasis progression, potentially through TNF-α signaling 204. TDEs (e.g., miR-934/203a-3p) induce macrophage M2 polarization via PTEN/PI3K/AKT pathway activation, synergizing with CXCL12/CXCR4 signaling to promote colorectal cancer liver metastasis 45, 205, 206. Additionally, the high permeability and lack of tight junctions in liver sinusoidal endothelial cells (LSECs) enable the liver to more effectively filter TCs from the bloodstream. For instance, the fenestrated structure of LSECs allows them to inhibit the activation of hepatic stellate cells under normal conditions, thereby maintaining the homeostasis of the liver environment. However, TCs tend to induce the defenestration of LSECs, leading to enhanced TC adhesion. These structural changes provide a more favorable environment for the growth of TCs 207. Notably, hepatic PMN formation is modulated by external factors, including gut bacteria, diet, and alcohol, which collectively shape immunosuppressive microenvironments 208-210.

### Brain

The formation of brain PMNs involves unique mechanisms due to the presence of the blood-brain barrier (BBB). TDEs regulate BBB permeability to orchestrate the spatiotemporal evolution of the brain PMN. Recent studies revealed that small-cell carcinoma-derived miR-374a-5p enhances BBB permeability by targeting γ-adducin and disrupting the distribution of ZO1 and occludin 129. Cytoskeletal remodeling drives tumor transendothelial migration, where TTLL4-mediated glutamylation of β-tubulin promotes the transport of multiple vesicular bodies, enhancing breast cancer cell adhesion to the BBB endothelium 211. TCs upregulate adhesion molecules such as ICAM1 and β3-integrin to strengthen their anchorage to the BBB endothelium and induce endothelial apoptosis, facilitating brain PMN formation 212. Notably, single-cell sequencing revealed CD276 upregulation in metastatic ECs, highlighting the unique immune checkpoint regulatory properties of the BBB 213. The abundance of microglia in the brain is important for the establishment of the PMN. Microglia-derived exosomal miR-19a recruits myeloid cells via CCL2 activation by suppressing PTEN, whereas endothelial-derived Dkk-1 induces M1-to-M2 microglial polarization, synergistically fostering an immunosuppressive microenvironment 214, 215. Targeted interventions (e.g., ESTA blocking the E-selectin/CD44 interaction) significantly inhibit breast cancer brain metastasis, underscoring the therapeutic potential of endothelial-specific targets 216, 217. Exogenous stimuli, such as nicotine exposure, accelerate brain PMN maturation by inducing TC metabolic reprogramming and enhancing stemness 218. These findings reveal that the BBB finely regulates the cascade of brain metastases through the dual mechanisms of structural remodeling and immunomodulation.

### Bone

Owing to their abundant blood supply and marrow microenvironment, bone tissue allows cancer cells to infiltrate and proliferate via hematogenous or lymphatic dissemination. Bone remodeling and homeostasis involve crosstalk between osteocytes and ECs in the marrow. Osteocytes promote angiogenesis by secreting VEGF to activate ECs 219^,^ while the RANKL/OPG balance regulates endothelial permeability to influence metastatic efficiency: elevated RANKL/OPG ratios increase vascular permeability, facilitating bone infiltration 220-222. Mechanical loading suppresses vascular permeability and PMN formation by reducing the RANKL/OPG ratio via fluid shear stress-induced inhibition of MMP9 secretion and weakened tumor-endothelial adhesion 223. Tumor-derived miR-135b enhances angiogenesis under hypoxia through the HIF-FIH pathway, synergizing with hypoxic microenvironments to accelerate bone metastasis 140, whereas siRNA nanodelivery systems targeting the bone marrow endothelium offer novel strategies for PMN intervention 224.

Osteoblasts and osteoclasts critically contribute to PMN formation. Cadherin 11 and integrin α5 mediate specific recognition of tumor-derived EVs by osteoblasts, creating PMNs that promote RUNX2-high breast cancer cell colonization 225. Concurrently, β2-adrenergic receptor activation in osteoblasts triggers VEGF-dependent angiogenesis, accelerating tumor colonization 226. RSPO2 and RANKL signaling through LGR4 recruits osteoclasts to remodel the bone matrix microenvironment, facilitating PMN development 227.

**Figure 3:**
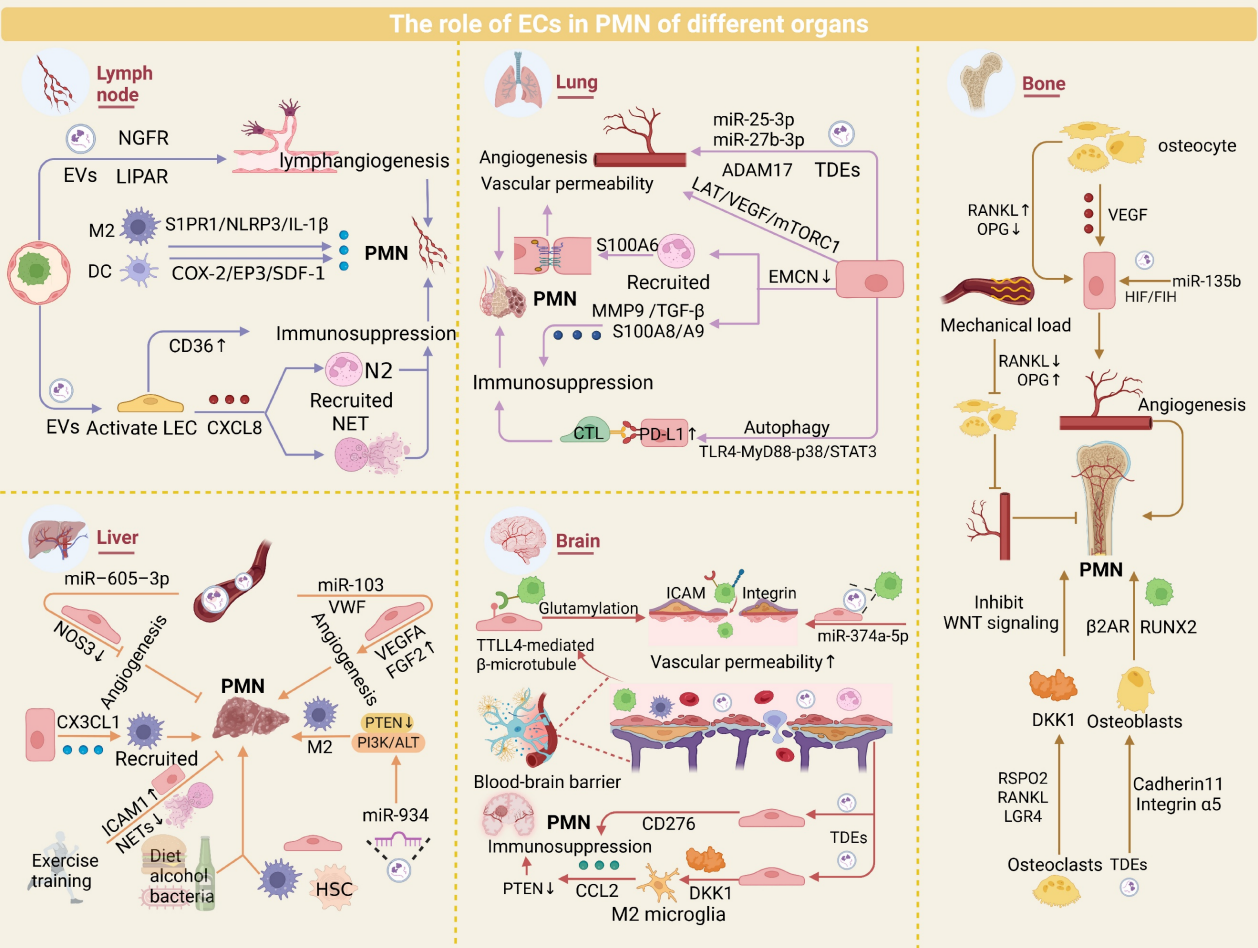
** Role of ECs in organ-specific PMN.** ECs play pivotal roles in PMN formation across major metastatic organs, including lymph nodes, lungs, liver, brain, and bone, by orchestrating angiogenesis and modulating immunosuppressive microenvironments. While these conserved EC-driven mechanisms underpin PMN development, organ-specific EC adaptations—such as BBB remodeling in the brain or sinusoidal fenestration regulation in the liver—further fine-tune metastatic tropism. Elucidating the shared and distinct mechanisms of EC-mediated PMN regulation provides critical insights into organotropism.

In summary, ECs exhibit common regulatory mechanisms and organ-specific functions in the process of tumor metastasis. In major metastatic organs such as lymph nodes, lungs, liver, brain, and bones, ECs generally drive the formation of PMNs by promoting angiogenesis and secreting exosomes to modulate the immune microenvironment. Meanwhile, ECs in each organ have unique roles. In lymph nodes, ECs enhance the stealthiness and invasiveness of TCs through lymphangiogenesis and high endothelial venous remodeling; in the lungs, the dense branching of the capillary network and the slow blood flow characteristics make CTCs more likely to be retained at the endothelial interface; in the liver, TCs induce the fenestration loss of LSECs, thereby enhancing the adhesion capacity of TCs; in the brain, ECs promote the infiltration of TCs by dismantling the “protective net” of the BBB (using certain small molecules to disrupt the barrier structure) and upregulating immune checkpoints; in bones, ECs regulate vascular permeability through the RANKL/OPG dynamic balance, providing a favorable growth environment for TCs. In conclusion, the commonalities of ECs in different metastatic organs provide the foundation for tumor metastasis, while the organ-specificity of ECs endows TCs with “niche selectivity” (Figure 3).

## Strategies and recent advances in targeting EC

### Targeting TDEs: Molecular Interventions in EC-Mediated PMNs

In recent years, interest in the role of TDEs in promoting the formation of the PMN through their interaction with ECs has increased (Table 1), highlighting the potential of targeting exosomes in the regulation of the PMN 228. Several drugs that inhibit the release of TDEs to impede cancer progression have been identified. For example, cannabidiol suppresses TDE release, potentially through alterations in mitochondrial function 229. TDE inhibitors such as chloramidine and bisindolylmaleimide-I have also been shown to increase the efficacy of chemotherapeutic agents 230.

**Figure 4:**
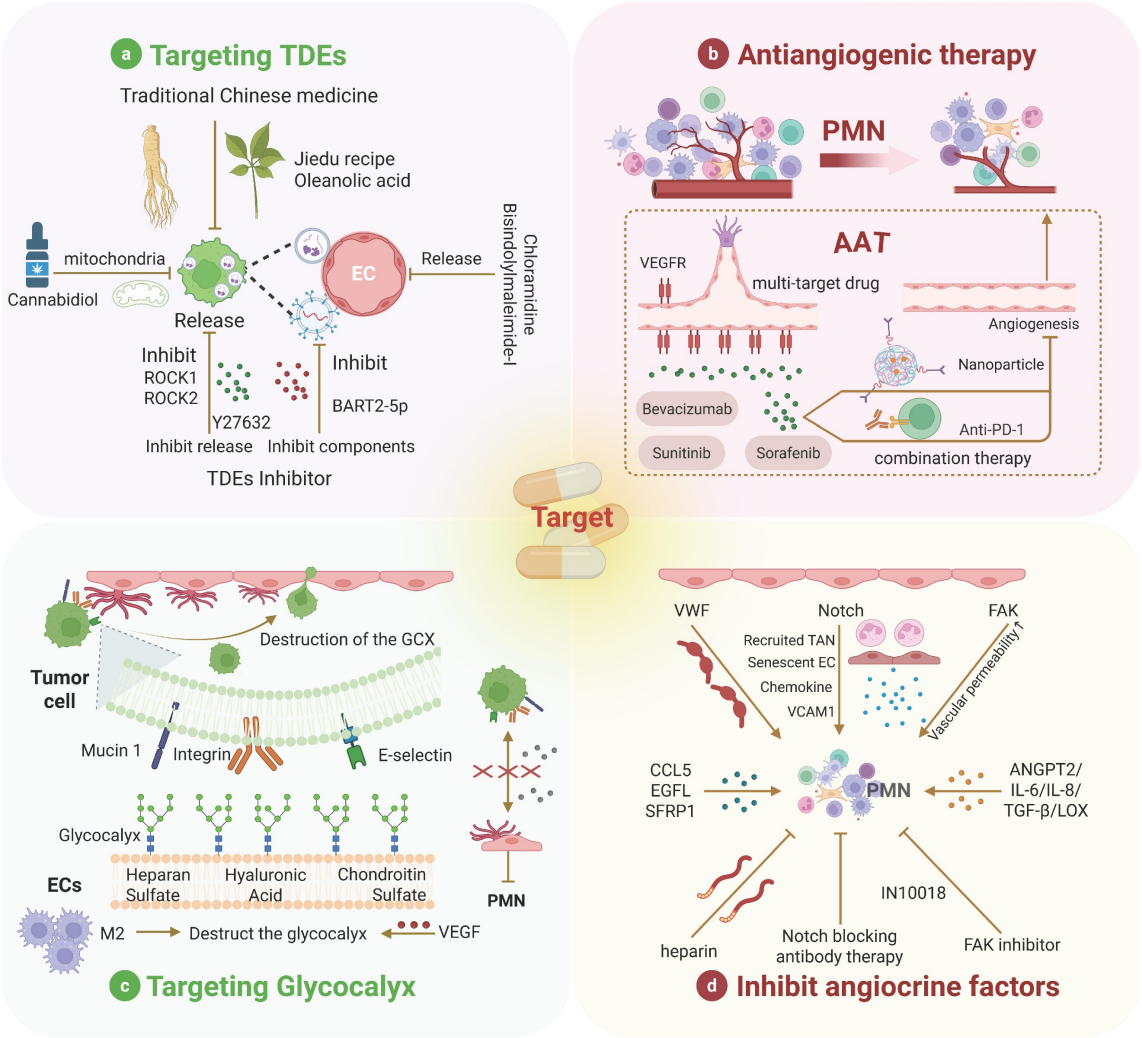
** Therapeutic strategies for targeting ECs in PMN.** Many therapeutic strategies for PMN have been proposed, with most targeting various cells within the PMN still in the developmental stage. Among these, we summarize four common therapeutic strategies targeting ECs. Parts a and c are preclinical therapies, and parts b and d are clinical therapies.

Additionally, Y27632, an inhibitor of ROCK1 and ROCK2, can block proteins involved in cell motility, thereby reducing TDE release 231. On the other hand, traditional Chinese medicine is also emerging as a potential method to inhibit TDE release 232. Recently, Jia et al. elucidated the mechanisms by which the Jiedu recipe and oleanolic acid inhibit PMN formation, which is associated with TDE release 233, 234. Another approach to targeting TDEs involves the inhibition of exosome-related components. For example, TDEs containing BART2-5p promote metastasis by inducing pyroptosis in ECs, and BART2-5p inhibitors can attenuate this effect 93. In addition to pharmacological inhibition, genetic manipulation is another extensively studied strategy for targeting and inhibiting TDEs. Disruption of genes regulating TDE biogenesis and secretion using RNAi and CRISPR-Cas9 systems has achieved TDE inhibition. For instance, RNA interference screening of 23 components of the endosomal sorting complex required for transport in MHC II-expressing HeLa-CIITA cells revealed that silencing HRS, STAM1, and TSG101 reduced the secretion of CD63 and MHC II associated with exosomes 235. However, this strategy still faces some adverse effects and challenges. Genetic manipulation may lead to off-target effects, such as unintended gene insertions, deletions, or mutations, thereby causing safety issues 236. Moreover, the current inhibition strategies have limited specificity, as they block both TDEs and non-TDEs, potentially inducing adverse reactions in tumor treatment. On the other hand, since TDE biogenesis involves multiple signaling pathways, single-target blockade can be easily weakened by compensatory mechanisms 237. Additionally, the high heterogeneity of exosomes in the bloodstream makes specific identification difficult 228. Therefore, it is necessary to develop multitarget pharmacological inhibitors. Meanwhile, to minimize side effects caused by non-TDEs, precise release of TDE inhibitors is also required (Figure 4a).

### Antiangiogenic therapies: Reducing pathways for tumor metastasis

PMN formation relies on endothelial “bridging” functions; inhibiting angiogenesis disrupts tumor intravasation, circulation, extravasation, and distant colonization while impairing immune cell recruitment for the establishment of an immunosuppressive niche 238. Anti-angiogenic therapies primarily target the VEGF signaling pathway to suppress tumor angiogenesis. Multiple kinase inhibitors (e.g., sorafenib and sunitinib) block VEGF/PDGF signaling and are widely used to curtail tumor progression by preventing bypass pathway activation 239. Paradoxically, sunitinib promotes PMN formation in metastatic breast cancer by inducing EC senescence, chemokine secretion, and cell junction loosening 240, necessitating cautious clinical application. Nanoparticle-based strategies enhance antiangiogenic efficacy 241. Apatinib-loaded nanoparticles inhibit tumor dissemination via VEGF/VEGFR2 blockade 242. In addition, antiangiogenic immunotherapy, which can increase the sensitivity of tumors to angiogenic therapy, has been a popular research direction in recent years 243. Preclinical evidence, such as the use of the bispecific antibody to jointly block ANGPT2 and VEGFA, can significantly improve antitumor immunity. Clinically, the combination of PD-1 and VEGF2 inhibitors for the treatment of HCC outperforms monotherapy in clinical trials 244. Currently, drug resistance remains the main challenge faced by AAT, with complex and diverse resistance mechanisms 245. For instance, bevacizumab, an anti-VEGFA antibody, has been approved for the treatment of various advanced metastatic cancers, including lung, colorectal, renal, breast, and recurrent glioblastoma. However, many patients treated with VEGF inhibitors, especially when combined with chemotherapy, may initially survive longer but eventually succumb to their disease due to the development of resistance. After inhibition of VEGF, ECs maintain survival through alternative signaling pathways such as Angiopoietin/Tie2, FGF, and Notch 246-251. In addition, multiple studies have shown that tumors escape therapeutic pressure by activating alternative angiogenic patterns, including intussusceptive angiogenesis, vessel co-option, and vasculogenic mimicry 252-254. In the future, exploring more effective AAT combination therapy regimens, developing specific biomarkers, and accurately grasping the “timing window” for treatment can significantly improve the clinical application of AAT 255. For example, the CXCR4 inhibitor AMD3100 can only effectively inhibit angiogenesis by blocking SDF-1-mediated precursor recruitment early after radiotherapy 256, 257. Alternative approaches, such as vascular promotion and vascular disruption, remain exploratory 258-260 (Figure 4b).

### Targeting endothelial GCX: structurally targeted intervention for ECs in the PMN

GCX and adhesion molecules regulate endothelial-tumor/immune cell interactions, modulating adhesion and permeability to inhibit metastasis. Previously, hemodynamic shear stress triggered GCX degradation, facilitating TC homing to ECs 261. Further studies have indicated that TCs can alter the endothelial GCX to form adhesion sites, thereby enhancing their ability to extravasate into surrounding tissues. This manipulation of the GCX is a crucial step in the metastatic process, suggesting that therapeutic strategies targeting these interactions may be feasible 262. Okorafor et al. focused on the impact of the physical environment on the extravasation of triple-negative breast cancer, emphasizing that the endothelial GCX acts as a barrier regulating this process. They proposed that understanding the physical mechanisms underlying these interactions could help identify new therapeutic targets to prevent metastasis 263. VEGF differentially reorganizes heparan sulfate and hyaluronic acid in ECs vs. TCs, creating proadhesive niches 264, 265. Fu et al. conducted an in-depth analysis of the effects of VEGF on the endothelial GCX in the context of the BBB. Their research indicates that while VEGF reduces GCX coverage on ECs, it increases GCX coverage on malignant breast cancer cells. This differential effect may facilitate the adhesion and migration of TCs across the BBB 264. Recently, Shi et al. provided a detailed map of the composition and structure of the GCX layer in the aged brain endothelium and revealed the significant impact of its dysregulation on BBB integrity and brain health 266. GCX degradation by macrophage-derived factors was shown to promote PMN maturation, whereas macrophage depletion preserves GCX integrity 97.

Disrupting hyaluronic acid‒CD44 interactions (key for TC‒EC adhesion) significantly reduces TC extravasation 262, 267, 268. GCX also modulates TDE uptake/release to drive angiogenesis and metastasis 269. However, research on endothelial GCX is mostly based on animal models or cultured cells, with more studies remaining in the preclinical stage, which cannot accurately reflect the human condition. In summary, understanding the mechanisms that regulate the interactions between ECs and TCs, as well as their responses to the physical environment and factors such as VEGF, is crucial for developing targeted therapies to inhibit metastatic progression 263 (Figure 4c).

### Inhibiting angiocrine signaling: blocking the tumor-promoting effects of EC

The concept of angiocrine signaling has evolved from the traditional understanding of ECs as mere participants in angiogenesis to their more complex role in controlling tumor metastasis 270. ECs express membrane-bound and secreted factors that influence tumor progression 108. For example, VWF, an angiocrine factor, has been demonstrated to enhance TC adhesion and transendothelial migration. The use of low-molecular-weight heparin to negatively regulate VWF secretion can inhibit tumor metastasis 271, 272. The Notch signaling pathway plays a central role in angiocrine regulation during tumor development, with sustained Notch1 activity inducing EC senescence and the expression of chemokines and adhesion molecules such as VCAM1, thereby promoting metastasis 39, 273. Treatment with Notch1- or VCAM1-blocking antibodies can prevent Notch-driven metastasis. Moreover, FAK in ECs has been identified as a major regulator of chemosensitivity in cancer therapy, with FAK inhibition reducing metastasis following gemcitabine treatment 274, 275. On the other hand, the role of TGF-β signaling has also been emphasized in advanced tumors. It has been observed that endogenous TGF-β signaling can promote TCs to evade inhibitory effects, which suggests that blocking this pathway may enhance the efficacy of anti-tumor therapies 276. This is in line with the finding that some signaling pathways have dual roles, and inhibiting these pathways can reduce tumor growth and alter immune responses. For instance, in colorectal cancer, inhibiting specific molecules such as ADAM17 and soluble JAGGED-1 is associated with the disruption of angiocrine signaling, further supporting the view that targeting these pathways can mitigate tumor-promoting effects 277, 278. EGFL7, which is associated with the ECM, is linked to primary tumor growth, angiogenesis, tumor metastasis, and drug resistance, highlighting the multifaceted role of angiocrine factors in cancer progression 279. Recently, Sfrp1 derived from TECs was shown to support cancer stem cell maintenance through WNT signaling, further emphasizing the complex interplay between TECs and TCs 280. While combinatorial targeting of angiocrine factors with established therapies has demonstrated clinical promise, the precise mechanisms underlying these agents' vascular remodeling effects necessitate further in vivo validation. Additionally, the spatiotemporal heterogeneity of angiocrine signaling within solid tumors and tumor-type specificity of individual angiocrine factors remain formidable challenges requiring multi-omics characterization 270. Overall, angiocrine factors are involved in various aspects of cancer progression, including proliferation, stemness, EMT, invasion, and immune suppression, making them promising platforms for developing effective therapeutic strategies (Figure 4d).

## Conclusion and Future Perspective

The PMN hypothesis is an emerging concept concerning tumor metastasis, primarily involving changes in vascular permeability, activation of stromal cells, remodeling of the ECM, and recruitment of immune cells. Its importance in cancer metastasis is increasingly recognized. In this study, we thoroughly discuss the complex relationship between the PMN and ECs, highlighting the crucial role of ECs in tumor metastasis and PMN formation. Additionally, we further synthesized current strategies for targeting ECs within the PMN, ranging from exosome inhibition to GCX modulation and angiocrine signaling blockade.

However, research gaps persist. While myeloid cells (e.g., macrophages and neutrophils) dominate PMN studies, EC-centric investigations remain underrepresented. Nevertheless, the role of ECs in tumor metastasis should not be overlooked, as their interactions with tumor and immune cells are crucial for understanding the mechanisms underlying tumor metastasis. Additionally, many therapeutic approaches, although promising in preclinical models, face translational challenges, particularly in clinical validation.

Future efforts should prioritize combinatorial therapies integrating EC-targeted interventions with immune modulation or chemotherapy to enhance efficacy. For example, combining AAT with immune checkpoint inhibitors can induce tumor vessel normalization, improve immune cell infiltration and function, and thus achieve a synergistic antitumor effect. Evaluating whether the combination therapy is synergistic or additive, as well as shifting the focus of antiangiogenic drugs from VEGF/R to other candidates (e.g., FGF/R), can help further optimize antiangiogenic immunotherapy. Moreover, using cutting-edge technologies such as single-cell transcriptomics and spatial transcriptomics can provide in-depth insights into the interactions between endothelial and immune cells, revealing their dynamic changes and functional differences in the PMN. These technologies can also help us revolutionize our understanding of PMN cellular heterogeneity. For example, macrophages and neutrophils exhibit diverse functional subsets within the PMN, the complexity of which is now resolvable at single-cell resolution. Leveraging these tools will clarify EC communication networks with PMN components (e.g., immune cells and the ECM) and unveil novel drivers of metastasis.

## Figures and Tables

**Table 1:** Mechanisms of TDEs targeting ECs to promote PMN formation.

Exosome composition	Primary tumor	Function and mechanism	Target Organ	Refs
**miR-BART2-5p**	NPC	Induces EC pyroptosis and increases vascular permeability by regulating MRE11A	Bone/Liver/Lung	124
**circ_0011496**	HCC	Circ_0011496 interacts with miR-486-5p to enhance angiogenesis and vascular permeability via the VEGF route	Lung	125
**circ-ZNF609**	ESCC	Circ-ZNF609 disrupts EC tight junctions via miR-150-5p/VEGFA and HuR/ZO-1 pathways	Liver/Lung/Lymph node	126
**miR-103a-3p**	NPC	Promoting TC proliferation and vascular permeability by targeting ZO-1 and ACOX-1	Lung/Lymph node	127
**miR-1270**	BC	Disrupts EC tight junctions by down-regulating ZO-1 and occludin expression	Lung	128
**miR-27b-3p**	CRC	Post-transcriptional expression of VE-Cad and p120 was inhibited by targeting their 3'-UTR in ECs.	Liver/Lung	92
**miR-374a-5p**	NSCLC	Regulates the distribution of ZO1 and occludin in ECs by targeting γ-adducin, increasing vascular permeability	Brain	129
**miR-605-3p**	GC	By regulating the secretion of NOS3, it can increase the NO level of EC and promote angiogenesis.	Liver	7
**MFI2-AS1**	NSCLC	Increased expression of NFAT5 through adsorption of miR-107, thereby activating the PI3K/AKT pathway and promoting angiogenesis	Lung	130
**miR-455**	NPC	Disrupts EC tight junctions and increases vascular permeability by targeting ZO-1	Lung	131
**miR-519a-3p**	GC	Targeting DUSP2 induces macrophage M2 polarisation and M2 macrophages promote angiogenesis	Liver	132
**miR-3157-3p**	NSCLC	By regulating the expression of VEGF/MMP2/MMP9 and occludin	Lung/Bone	133
**miR-638 /miR-663a/** **miR-3648 /miR-4258**	HCC	By down-regulating VE-cadherin and ZO-1 in ECs	Lung/Lymph node/Bone	134
**miR-1260b**	NSCLC	Inhibition of HIPK2 in ECs promotes angiogenesis and enhances tumour cell migration and drug resistance	Lung	135
**miR-619-5p**	NSCLC	Inhibition of RCAN1.4 promotes angiogenesis and tumour metastasis	Lung	136
**miR-103**	HCC	Inhibition of VE-Cadherin, p120-catenin, and ZO-1 expression disrupts EC tight junctions and adhesion junctions	Liver/Lung	137
**miR-25-3p**	CRC	Regulation of VEGFR2, ZO-1, occludin and Claudin5 expression in ECs by targeting KLF2 and KLF4	Liver/Lung	93
**miR-23a**	LC	Enhancement of angiogenesis and vascular permeability by targeting PHD1, PHD2, and ZO-1	Lung	138
**miR-105**	BC	Disrupts EC tight junctions by targeting ZO-1	Bone/Lung/Brain	139
**miR-135b**	MM*	Enhancement of tube formation in ECs under hypoxic conditions via the HIF-FIH signalling pathway	Bone	140
**miR-181b/ miR-27a/** **miR-484/ miR-324-3p**	RCC	Provision of pro-angiogenic mRNAs and miRNAs	Lung	141
**miR-126**	BC	Targeting IGFBP2, PITPNC1, and MERTK to inhibit EC recruitment and angiogenesis	Lung/Bone	142
**miR-9**	BC	Down-regulation of E-cadherin, activation of the β-catenin/VEGF pathway, and promotion of angiogenesis	Lung	143
**CLTA**	HCC	Stabilisation and up-regulation of BSG in ECs to remodel the pre-metastatic microvascular niche	Lung/Lymph node/Bone	144
**vWF**	HCC	Promoting angiogenesis and metastasis by facilitating the formation of a positive feedback loop between tumours and ECs by VEGF-A and FGF2	Lung	145
**Tspan8-α4/β1** **Tspan8-α6/β4**	MT	Activation of EC membrane receptors induces activation of signalling pathways and up-regulation of transcription factors to promote angiogenesis.	Lung	146
**ADAM17**	CRC	Regulates membrane localisation of VE-cadherin and enhances vascular permeability by targeting ECs	Liver/Lung/Peritoneum	94
**NDPK-B**	BC	Promotes EC migration and disrupts monolayer integrity, leading to vascular leakage in the lungs	Lung	147
**NID1**	HCC	Enhancement of angiogenesis and pulmonary vascular endothelial permeability	Lung	148
**uPAR**	MM	Binds to ECs and activates VE-Cadherin, EGFR, and uPAR expression	Skin/Lymph node/Lung	149
**ErbB2/** **CRK**	BC*	Promoting EC proliferation and invasion and increasing vascular permeability through FAK and PI3K/AKT signalling pathways.	Lung/Liver/Bone	150
**TF**	BC/PC	Activation of PAR-1 on EC causes upregulation of E-selectin expression and IL-8 secretion	Peritoneum/Liver/Bone	151

*Abbreviation: NPC: Nasopharyngeal carcinoma, HCC: Hepatocellular carcinoma, ESCC: Esophageal squamous cell carcinoma, BC: Breast cancer, CRC: Colorectal cancer, NSCLC: Non-small cell lung cancer, GC: Gastric cancer, LC: Lung cancer, MM*: Multiple myeloma, RCC: Renal cell carcinoma, MT: Mouse Tumor, MM. Melanoma, BC*: Bladder cancer, PC: Pancreatic cancer.

## Abbreviations

PMN: Premetastatic niche
EC: Endothelial cell
TC: Tumor cell
CTCs: Circulating tumor cells
EV: Extracellular vesicle
TDE: Tumor-derived exosome
ECM: Extracellular matrix
MDSC: Myeloid-derived suppressor cell
TDSF: Tumor-derived secretory factor
TME: Tumor microenvironment
NET: Neutrophil extracellular trap
TAN: Tumor-associated neutrophil
EMT: Epithelial-mesenchymal transition
FAK: Focal adhesion kinase
TAM: Tumor-associated macrophage
Treg: Regulatory T cell
HIF: Hypoxia inducible factor
TRAIL: Tumor necrosis factor-related apoptosis-inducing ligand
BBB: Blood-brain barrier
GCX: Glycocalyx
LSEC: Sinusoidal endothelial cell
AAT: antiangiogenic therapy
